# Systematic method for developing tailored strategies for implementing point-of-care procalcitonin testing to guide antibiotic prescribing in Swiss primary care: a protocol for a mixed-methods participatory approach

**DOI:** 10.1136/bmjopen-2024-091285

**Published:** 2025-03-05

**Authors:** Aline Wolfensberger, Sophie CL Gendolla, Jelena Dunaiceva, Catherine Plüss-Suard, Anne Niquille, Anna Nicolet, Joachim Marti, Byron J Powell, Rahel Naef, Noémie Boillat-Blanco, Yolanda Mueller, Lauren Clack

**Affiliations:** 1 Institute for Implementation Science in Health Care, University of Zurich, Zurich, Switzerland; 2 Department of Infectious Diseases and Hospital Epidemiology, University Hospital Zurich, Zurich, Switzerland; 3 Department of Family Medicine, Unisanté, Centre for Primary Care and Public Health & University of Lausanne, Lausanne, Switzerland; 4 Swiss Centre for Antibiotic Resistance (ANRESIS), Institute for Infectious Diseases, University of Bern, Bern, Switzerland; 5 Department of Ambulatory Care, Unisanté, Centre for Primary Care and Public Health & University of Lausanne, Lausanne, Switzerland; 6 Institute of Pharmaceutical Sciences of Western Switzerland, University of Lausanne & University of Geneva, Lausanne, Switzerland; 7 Department of epidemiology and health systems, Health Economics and Policy Unit, Unisanté, Centre for Primary Care and Public Health & University of Lausanne, Lausanne, Switzerland; 8 Center for Mental Health Services Research, Brown School, Washington University in St. Louis, St. Louis, Missouri, USA; 9 Center for Dissemination and Implementation, Institute for Public Health, Washington University in St Louis, St Louis, Missouri, USA; 10 Center of Clinical Nursing Science, University Hospital Zurich, Zurich, Switzerland; 11 Infectious Diseases Service, Department of Medicine, Lausanne University Hospital, Lausanne, Vaud, Switzerland

**Keywords:** Primary Care, Protocols & guidelines, Implementation Science

## Abstract

**Abstract:**

**Introduction:**

Antimicrobial resistance is a major global health threat, driven largely by the misuse and overuse of antibiotics. Point-of-care (POC) tests for inflammatory biomarkers like procalcitonin (PCT) have shown promise in reducing unnecessary antibiotic prescriptions. The hybrid type II ImpPro trial aims to evaluate the implementation and effectiveness of POC-PCT on antibiotic prescriptions by primary care physicians (PCP) in French-speaking Switzerland. Implementation is planned to include a multifaceted strategy delivered mainly, but not exclusively, via PCP quality circles. Currently, little guidance exists on how to best tailor the implementation strategies to a specific context. This study protocol describes the comprehensive approach taken within ImpPro to develop a multifaceted and multilevel strategy for POC-PCT implementation.

**Methods and analysis:**

Our mixed-methods participatory implementation research study consists of four phases: (1) determinant identification; (2) determinant prioritisation; (3) implementation strategy ideation and (4) implementation strategy selection and refinement. All phases of the study will be guided by well-established implementation theories, models and frameworks. For 1, to identify the possible barriers and facilitators for implementation, we will conduct semistructured interviews with stakeholders followed by deductive coding using the updated Consolidated Framework for Implementation Research and inductive thematic analysis. In 2, to identify the key determinants, we will conduct online focus group discussions and vote on the importance and changeability of determinants. In 3, we will conduct interviews and an expert brainstorming session, followed by deductively coding implementation ideas according to the Expert Recommendations for Implementing Change compilation. In 4, we will conduct focus group discussions with experts and stakeholders about the APEASE criteria (ie, affordability, practicability, (cost-)effectiveness, acceptability, side effects and safety and equity) of these strategies, followed by a rapid data analysis approach to select the implementation strategies.

**Ethics and dissemination:**

This study does not fall within the scope of the Human Research Act, and the necessity for a formal evaluation was waived from the Cantonal Ethics Committee (Req-2023–00392). The results of our study will be shared among the Antimicrobial Stewardship in Ambulatory Care Platform network, published in peer-reviewed scientific journals, and will be presented at international and national conferences.

**STRENGTHS AND LIMITATIONS OF THIS STUDY**

- This study employs explicit and transparent phases to tailor a multifaceted point-of-care-procalcitonin (POC-PCT) implementation strategy, integrating established frameworks and tools with intuitive approaches.
- This study actively engages stakeholders and uses purposive and stratified sampling to ensure diverse stakeholder representation.
- Stakeholder interviews and workshops may be biased towards highly motivated, opinionated, outspoken, technology-literate participants.
- There is limited opportunity for pilot testing in the current study, so implementation strategies will be evaluated and refined during the ImpPro project for a broader roll-out of POC-PCT.

## Background

 Antimicrobial resistance was declared by the WHO as one of the top 10 global health threats facing humanity.^1^ Misuse and overuse of antimicrobials are the main drivers in the development of drug-resistant pathogens.^1^ In Switzerland, 85% of antibiotics are prescribed in the outpatient setting, with 20% for lower respiratory tract infections.^2^ The prescriptions are higher in the French/Italian-speaking part of Switzerland than in the German-speaking part.^2^ Antimicrobial stewardship (AMS) refers to a set of coordinated strategies and activities designed to optimise the use of antibiotics. One specific AMS intervention is the introduction and utilisation of point-of-care (POC) tests, such as those for inflammatory markers like C-reactive protein (CRP) or procalcitonin (PCT). Together with a clinical assessment, such tests can help to reduce diagnostic uncertainty and promote informed clinical decisions about whether antibiotic therapy is indicated. While CRP-POC is already used by Swiss primary care physicians (PCP), PCT-POC is not yet reimbursed (and thus not used). Recently, UltraPro, a cluster randomised trial conducted in 60 primary care practices in Switzerland, showed that POC-PCT reduces antibiotic prescriptions by an absolute 26% (95% CI −41% to −10%) in patients with clinical pneumonia, without affecting patients’ safety.^3^

In anticipation of the future reimbursement of POC-PCT in the outpatient setting, the ImpPro hybrid-type II effectiveness implementation trial—a successor of the UltraPro trial—aims to understand whether the implementation of POC-PCT in the general population of PCPs in the French-speaking part of Switzerland leads to the adoption (the intention or decision to employ), implementation (the purposeful actions taken to put into use) and continued utilisation (the ongoing use) of POC-PCT and a decrease in antibiotic prescriptions for lower respiratory tract infections. Implementation is planned to primarily happen via quality circles, that is, collaborative groups of physicians or interprofessional groups of pharmacists and physicians that systematically identify and address issues related to patient care with the aim of enhancing overall service quality and efficiency.^4^ The ImpPro research project is designed as a cluster randomised trial, with clusters being PCPs assigned to the same quality circle.

The outpatient healthcare system in Switzerland can be described as complex: it is federally organised, with a high degree of decentralisation, and includes a multitude of relevant stakeholders (eg, PCP, patients, quality circle moderators, various professional organisations, insurance companies, laboratory machine providers and health authorities). Literature about the implementation of POC tests in complex systems is scarce, and most AMS interventions have been conducted in a research setting.^5^ Avent *et al* argue that not only a bottom-up but also a top-down approach is needed to implement AMS interventions in the primary care setting.^5^ Implementing POC-PCT in the French-speaking part of Switzerland seeks to address the individual behaviour of general practitioners, but it likely will need to be supported by a broad-scale system change.

In implementation or quality improvement projects, the implementation strategies constitute the ‘active ingredients’. Implementation strategies are the methods and techniques used to support individual and organisational change to enhance the adoption, implementation and sustainability of an intervention.^6^ Tailored implementation was described as a ‘prospective process involving the (1) identification and prioritisation of determinants likely to influence the implementation and (2) selection, operationalisation and application of implementation strategies likely to address the identified determinants’.^7^ Although evidence about the benefits of tailoring implementation strategies to the local context is limited,^8^ the application of strategies that are purposefully chosen to address identified barriers is intuitive and frequently recommended.^9^ The process leading to the selection of the most appropriate implementation strategies is a complex task, and although some general guidance exists from the literature,^10 11^ methods often have to be combined and/or adapted to fit a project’s needs. Still, there is a need to enhance methods for developing and tailoring implementation strategies.^12^

In this article, we present the methods through which the ImpPro study group will develop the multifaceted and multilevel implementation strategy for POC-PCT in the primary care setting of French-speaking Switzerland. Specifically, our objectives are to (1) identify determinants relevant for the adoption, implementation and continuing utilisation of POC-PCT in primary care practices and (2) design a multifaceted implementation strategy tailored to the identified contextual determinants that aims to increase adoption, implementation and continuing utilisation of POC-PCT. By detailing our methods, we aim to facilitate reproducibility for the researchers and clinicians in the related fields.

## Methods

### Study phases

**Figure 1:**
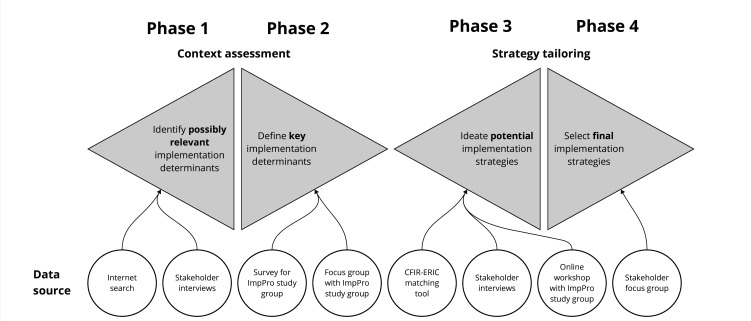
Tailored implementation strategy development process, Graphic representation of the four phases of the strategy development process, with the data sources that are used for each specific phase. CFIR, Consolidated Framework for Implementation Research; ERIC, Expert Recommendations for Implementing Change.

The study consists of four phases (figure 1), similar to the ‘Double Diamond’, a design process model developed by the British Design Council.^13^ In phase 1, ‘Determinant identification’, all possibly relevant implementation determinants will be identified. In phase 2, ‘Determinant prioritisation’, the identified implementation determinants will be prioritised based on relative importance and changeability to decide on key determinants that will be addressed in subsequent phases. In phase 3, ‘Implementation strategy ideation’, a broad range of potential implementation strategies will be ideated and conceptualised. Finally, in phase 4, ‘Strategy selection and refinement’, the strategies that exhibit the highest likelihood of effectively enhancing the adoption, implementation and continued utilisation of POC-PCT-guided antibiotic prescription will be chosen and specified.^6^ Phase 1 began in June 2023, and phase 4 is expected to be completed in June 2025.

### Guiding models and frameworks, supporting tools and methods

All phases of the study will be guided by well-established theoretical implementation science models, theories and frameworks outlined in table 1. First, the updated version of the Consolidated Framework for Implementation Research (CFIR)^14^ will be used as the overarching framework to guide data collection and analysis to identify implementation determinants. The reason for choosing CFIR as our guiding framework is due to its holism, as it covers constructs across the five major domains (ie, intervention characteristics, outer setting, inner setting, characteristics of the individuals and process of implementation), which are considered necessary to evaluate all relevant determinants in the Swiss outpatient system. The Expert Recommendations for Implementing Change (ERIC), providing a compilation of implementation strategies^15^ grouped into nine clusters by Waltz *et al*,^16^ will be used to categorise implementation strategies. Second, to develop and describe tailored implementation strategies, the CFIR–ERIC matching tool will be used to identify a prioritised list of implementation strategies that match the identified CFIR determinants.^17 18^ Third, the Implementation Research Logic Model (IRLM) will be used to specify the link between implementation determinants, strategies and outcomes.^19^ Proctor *et al*’s recommendations for specifying implementation strategies across seven dimensions (ie, actor, action, action targets, temporality, dose, implementation outcomes addressed and theoretical justification) will be employed to specify the implementation strategies in sufficient detail to ensure they could be replicated in research and practice.^6^ Last, to prioritise implementation determinants, we will apply the framework developed by Green and Kreuter, which evaluates each determinant’s changeability and importance,^20^ and to evaluate and prioritise implementation strategies, we will work with the APEASE criteria developed by Michie *et al*.^21^

**Table 1:** Models, theories and frameworks guiding the strategy development

Framework/theory	Type and description	Relevance for the ImpPro study	Adaptations for the ImpPro study
Consolidated Framework for Implementation Research (CFIR), updated Version^14^	The CFIR is one of the most highly cited frameworks in implementation science. It is a metatheoretical determinant framework, which provides a pragmatic structure to predict or explain barriers and facilitators for implementation effectiveness.^14^ While the CFIR framework was originally published in 2009,^33^ an updated version was published in 2022.^14^ It includes five domains: innovation, outer setting, inner setting, individuals and implementation process. Within these domains are 48 constructs and 19 subconstructs that can each act as a barrier and/or facilitator to the implementation of an intervention.	The CFIR framework includes a wide range of constructs that relate not only to individual characteristics and innovation characteristics but also to structural characteristics of the primary care practices and the healthcare system. Anticipating divers and relevant determinants from the outer setting, the CFIR provides the necessary constructs to describe and identify determinants from this domain.	As we are evaluating the context of a not-yet delivered implementation, the ‘implementation process domain’ of the updated CFIR will be amended to an ‘implementation process suggestions’ domain, and potential implementation strategies will be coded under this overarching theme (and will later be coded according to the ERIC compilation).Subconstructs will not be coded.
Expert Recommendations for Implementing Change (ERIC)^15^	The ERIC study aimed to refine a compilation of implementation strategy terms and achieved consensus on a final compilation of 73 implementation strategies.^15^ In a second stage, Waltz *et al* categorised these strategies into nine distinct groups: engage consumers; use evaluative and iterative strategies; change infrastructure; adopt and tailor to the context; develop stakeholder inter-relationships; use financial strategies; support clinicians; provide interactive assistance; train and educate stakeholders.^16^	The ERIC list of strategies will serve as building blocks for constructing multifaceted implementation strategies for the implementation of POC-PCT-guided antibiotic prescription. The comprehensive compilation allows using a systematic published terminology.	None.
Recommendations for specifying and reporting implementation strategies^6^	The recommendations for specifying and reporting implementation strategies, published in 2013, proposed guidelines for naming, defining and operationalising implementation strategies in terms of seven dimensions: the actor, the action, the action targets, the temporality, the dose, the implementation outcomes addressed and the theoretical, empirical or pragmatic justification. The aim was to improve the reporting of the implementation strategies in research studies.^6^	The definition of the dimensions for implementation strategies will be used to specify the dimensions of the potential and final selection of implementation strategies. This will enable the measurement of implementation fidelity and increase the reproducibility of the implementation strategies.	We will work with the dimensions ‘actor’, ‘action’ and ‘action target’. ‘Temporality’ and ‘dose’ will be described as deemed necessary and useful for stakeholders. ‘Theoretical justification’ and ‘implementation outcomes addressed’ will not be defined.
Implementation Research Logic Model (IRLM)^19^	The IRLM was created to specify the conceptual linkages between implementation determinants, implementation strategies and implementation outcomes. It aims to enhance the rigour and transparency of describing the often-complex processes of implementing evidence-based interventions in healthcare delivery systems.	The IRLM will be used to specify the linkage between the implementation determinants, strategies and desired outcomes. It will thus support the stakeholders in the selection process for implementation strategies.	None.
CFIR–ERIC Matching Tool^17^	The CFIR–ERIC matching tool is a tool developed based on the knowledge of 169 experts who were asked to choose strategies that would best address the specific CFIR barriers. The tool allows users to receive a prioritised list of strategies on specified CFIR barriers.	The CFIR–ERIC Matching Tool supports and complements the search for strategies to consider.	To apply the CFIR–ERIC Matching tool, the determinants coded with the updated version of CFIR^14^ will be recoded with the CFIR original version^33^.
APEASE criteria^21^	The APEASE criteria were published in 2014 and provide a method for systematically evaluating contextual determinants or implementation strategies. The acronym stands for acceptability, practicability, effectiveness, affordability, side effects and equity.	We will apply specific elements of the APEASE criteria to evaluate the ideated implementation strategies with relevant stakeholders regarding their applicability and effectiveness in the real-life context.	Only specific elements of the APEASE criteria will be evaluated, depending on the strategy and the stakeholder group. Likely, acceptability, practicability and effectiveness will be included in the evaluation.

ABantibioticPCTprocalcitoninPOCpoint-of-care

### Phase 1: determinant identification

#### Specific aim of phase 1

To produce a comprehensive list of potential determinants (barriers and facilitators) for adoption, implementation and continued utilisation of POC-PCT-guided antibiotic prescription in primary care practices in French-speaking Switzerland.

#### Literature review and stakeholder interviews

First, to identify and familiarise ourselves with potential stakeholders (individuals and organisations) relevant to POC-PCT testing in primary care practices in the French-speaking part of Switzerland, an internet search for governmental reports and homepages of relevant groups, organisations and commercial companies will be conducted. We anticipate gaining insights into the structural characteristics, dependencies and activities of these stakeholders, as well as their inter-relationship with PCP. Then, we will conduct semistructured interviews of 30–60 min with individual stakeholders, either in person or via video. The interview guide with open-ended questions will be based on the updated CFIR^14^ and will iteratively be adjusted to fit the role of each interviewee and to close information gaps identified in previous interviews. An exemplary interview guide is provided in the online supplemental file 1. Interviews will be conducted by a team of two researchers: the primary interviewer will lead the discussions, while the second researcher will take the role of a note-taker and may pose additional questions if needed. Interviews will be videotaped and/or audiotaped if written informed consent is given by the interviewee; alternatively, handwritten notes will be taken during the interview by the second researcher.

#### Participants

Interview participants will be chosen based on a purposive sampling technique, aiming to recruit the most relevant stakeholders for adoption, implementation and utilisation of POC-PCT. An estimated number of 20–40 interview partners will be included. We assume that PCPs, representatives from professional and political organisations serving and representing Swiss PCP, medical practice assistants, quality circle moderators and patient representatives will likely be interviewees. A snowball sampling technique will be applied, beginning with ImpPro research group members who have an overview of the stakeholder’s landscape due to their work and involvement in the UltraPro predecessor study and the abovementioned internet search. In the stakeholder group of PCPs specifically, an additional stratified sampling based on predefined criteria including gender, years of experience, location of practice (urban or rural area) and employment conditions (independent vs employed, group practice vs single practice) will be performed. Both sampling strategies aim at identifying individuals with excellent knowledge of the topic under study and, therefore, the capacity to provide high information power.^22^ Interviewees participating in an interview in their free time, like self-employed PCPs, will be compensated with a book voucher.

#### Data analysis

Interview recordings will be transcribed verbatim and then qualitatively analysed by combining deductive and inductive approaches. Deductive coding will be based on the domains and constructs of the CFIR. Topics not covered by the CFIR framework will be identified and coded inductively. A coding manual of determinants will be developed in an interactive way based on the first approximately 10 interviews and will then be applied to code the remainder of the transcripts. Data coding will be done by two researchers (SG and AW) using the software MaxQDA.^23^ Consensus about codes will be reached by discussion or by consultation of a third researcher (LC) in case of disagreement. The following inductive thematic analysis will allow for a more comprehensive and nuanced understanding of the data and will generate a list of all potentially relevant implementation determinants and determinant categories. Initial descriptive codes will be grouped together into categories in an iterative, collaborative manner. Data will continuously be revisited to ensure that the categories reflect all relevant information. Finally, overarching themes that meaningfully group determinants will be created in the same way.

### Phase 2: determinant prioritisation

#### Specific aim of phase 2

To establish the list of key implementation determinants for POC-PCT-guided antibiotic prescription, to be addressed in subsequent phases, drawing from the list of potential determinants identified in phase 1.

#### Expert survey and focus group discussions

A mixed-methods approach will be applied. First, determinants identified in phase 1 will be quantitatively evaluated for (1) importance and (2) changeability^20^ by questionnaire using the web-based survey tool Unipark.^24^ The ‘importance’ of a determinant is defined as the likelihood that addressing this determinant will significantly enhance the implementation of POC-PCT; the ‘changeability’ of a determinant is defined as the feasibility of modifying it with a reasonable investment and within the project’s timetable. To facilitate rating for the survey participants, the determinants will be complemented with contextual information from the interviews. Rating of both importance and changeability will be done on a Likert scale, with 1 as a minimum and 10 as a maximum value. In the qualitative part of the study phase 2, data will be discussed in an online focus group using Miro,^25^ a collaborative platform providing a virtual whiteboard. Implementation determinants and their importance and changeability ratings from the survey will be displayed graphically to facilitate discussion and provide some guidance. The displayed determinants and their survey ratings will be discussed, and additional information from the interviews will be provided by the interviewers if necessary. Determinants will be excluded if the focus group agrees that there is no need to address them or if they are not deemed changeable within the project’s timetable. This step is followed by the next quantitative prioritisation part, consisting of an online dot-voting regarding the importance of the remainder of determinants, which is now informed by insights from the additional information provided and the discussions within the group. Based on that, there will be an informed selection of key determinants that exhibit high-importance values, and that will be addressed in the subsequent phases. Screenshots of the whiteboard and recordings of the session will store data for analysis.

#### Participants

The survey will be answered by the members of the ImpPro study group, including a diverse group of 10–12 experts from the fields of primary care medicine, implementation science, infectious diseases, pharmacy and health economics. The focus group interview will be facilitated by one member of the ImpPro study group having a background in infectious diseases and implementation science (AW); participants will be selected among the ImpPro study group including about 6–10 people.

#### Data analysis

Survey data and online dot-voting data will be analysed by calculating the median and IQR of importance and changeability for each determinant.

### Phase 3: implementation strategy ideation

#### Specific aim of phase 3

To produce a wide selection of implementation strategies designed to increase the adoption, implementation and utilisation of POC-PCT, based on the key implementation determinants identified in phase 2.

#### Stakeholder interviews and workshops

Two methods will be used to ideate potential implementation strategies addressing the key determinants while targeting different stakeholder groups (eg, PCPs, representatives of organisations), and throughout all relevant implementation stages (ie, preparation, implementation and sustainment^26^). First, we will draw on data collected during the semistructured interviews described in phase 1, where one question was directed towards the ‘optimal implementation of POC-PCT’ and revealed the ideas and perceptions of interviewed stakeholders about a hypothetic implementation of POC-PCT. Second, a brainstorming session will take place during an online workshop using the collaborative platform Miro,^25^ which allows for grouping, sorting and rearranging strategy ideas. Strategies tailored to the key determinants identified in phase 2 will first be ideated by doing ‘brainwriting’ (a silent form of individual brainstorming). At this stage, to encourage creativity and reduce fear of failing, strategies will be outlined at a fundamental level, prioritising quantity over quality. Also, unconventional and diverse ideas will be embraced, fostering a broad range of strategic possibilities without the need for detailed refinement. Idea generation will be supported by providing the workshop participants with the top 5 implementation strategies identified by using the CFIR–ERIC Matching Tool. The workshop participants will then arrange the ideas into conceptually consistent piles targeting one or several determinants. Next, the participants will select the most promising strategies by online dot-voting. The selected strategies will be further elaborated by the workshop participants, to specify the actor (who enacts the strategy), the action (activities, steps and processes enacted), the action target (who or what the strategy affects), the temporality and the dose (when and how often the strategy is enacted), as well as the rationale according to the proposition of Proctor *et al*.^6^ The rationale for developing the specific strategy will be pragmatic at this stage and implicit by arranging the strategy next to the determinant it aims to address. Screenshots of the whiteboard and recordings of the session will be stored for subsequent data analysis.

#### Participants

The interview participants will consist of the same individuals as described in phase 1. Workshop participants will comprise members from the ImpPro study group described in phase 2.

#### Data analysis

Interview data as well as the implementation strategies elaborated in the brainstorming workshops will be deductively coded using the ERIC framework and the categories of Waltz *et al*.^15 16^ The IRLM will be used by the implementation scientists team (SG, AW and LC) to ensure each strategy has a coherent rationale and that conceptual linkages between implementation determinants, implementation strategies and implementation outcomes are clarified.^19^ At this point, implementation determinants deemed ‘not changeable’ will be included. Strategies without coherent rationale or made impossible by contextual factors will be excluded.

### Phase 4: implementation strategy selection and refinement

#### Specific aim of phase 4

To produce a final selection of implementation strategies likely to increase the adoption, implementation and continued utilisation of POC-PCT to guide antibiotic prescription in the primary care setting in French-speaking Switzerland.

#### Focus groups

Focus group meetings (in person or via video) will be held and facilitated by the members of the implementation science expert team. Potential implementation strategies from phase 3 will be grouped regarding stakeholders involved or targeted and discussed with the respective stakeholders regarding relevant aspects of the APEASE criteria (eg, affordability, practicability, effectiveness, acceptability, side effects and safety and equity).^21^ Any strategy dimension (eg, actor and temporality) can still be refined at this point and amended implementation strategies will be again discussed regarding their APEASE criteria. Interviews will be videotaped and/or audiotaped after written informed consent has been given from the participants. If necessary, visualisation of data will be facilitated using the platform Miro.^25^ If visual data is generated, this will be stored by screenshots.

#### Participants

Several focus groups, each with 3–10 participants, will be held as needed. Potential focus groups include PCPs, quality circle representatives and representatives from other organisations. Participants will discuss the implementation strategies involving or affecting their respective professional groups.

#### Data analysis

The data will be analysed by applying a rapid data analysis approach using a matrix organised by strategy and APEASE criteria, which will also focus the inquiry. During the focus groups, one implementation team member will guide the discussion, and the other will continuously document findings on the matrix. Both team members will additionally document findings afterwards when again listening to the audiotaped discussions. Results of the analysis will be incorporated in a last refinement and selection of the components of the final multifaceted implementation strategy. The components will be summarised, and linkages between implementation determinants, strategies and outcomes will be depicted by using the IRLM and presented to the ImpPro research team to obtain a final consensus.

## Discussion

POC-PCT is an AMS intervention with the potential to safely reduce antibiotic prescriptions for pneumonia.^3^ For Swiss PCP, it was shown that general practitioners who used POC-PCT within a randomised controlled trial had mostly positive attitudes towards the test, trusted the test result and perceived POC-PCT to increase their self-confidence and to facilitate communication with patients.^27^ Little is known, however, about other determinants for the adoption, implementation or utilisation of POC-PCT in the Swiss outpatient sector. Outside of Switzerland, data about POC-PCT is lacking as well, but some information is available for POC-CRP, an inflammatory biomarker similar to PCT: a qualitative study in English general practices found that costs, time, access to the POC machine, and the effects on clinical workflow were barriers for implementing POC-CRP,^28^ while a 2016 US study identified concerns about overuse, inaccuracy and integration into family medicine clinics’ workflows to be barriers for POC-CRP in the management of acute respiratory tract infections.^29^ For the ImpPro research project—a study aiming to investigate the implementation and effectiveness of POC-PCT—we aim to develop an implementation strategy that is tailored to the implementation context.

To develop this protocol, we relied on published literature about tailoring implementation strategies to the context,^17^,^19^ on know-how and experience within our implementation science project team (LC, AW and SG) and on input from experts outside the project team (RN and BP). From the identification of determinants to the final selection of implementation strategies, we applied a systematic, theory-informed approach. While the guiding theories, models and frameworks were chosen at the very beginning of the project, some of the tools and methods to identify key determinants and to tailor implementation strategies were iteratively selected based on the results of preceding phases and the project’s timeline constraints. This approach was necessary, given the challenge of predicting both the quantity and content of potentially relevant implementation determinants and strategies.

Our four-phase procedure resembles the ‘double diamond model’, a problem-solving framework developed by the British Design Council and a visual representation of the user- or human-centred design process.^13^ As other researchers, we view the process of tailoring strategies to the implementation context as a developmental process, requiring a systematic and iterative approach.^30 31^ We translated the four stages of the double diamond ‘discover’, ‘define’, ‘develop’ and ‘deliver’ to the four phases of ‘determinant identification’, ‘determinant prioritisation, ‘implementation strategy ideation’ and ‘implementation strategy selection and refinement.’ The iterative nature of tailoring is represented in the divergent and convergent four-phase procedure, and in the refinement of the implementation strategies within phase four, where stakeholder inputs will lead to adjustment of implementation strategies.

Even though carefully elaborated, our methods might have some important limitations. First, there could be a selection bias. We anticipate that physicians participating in individual interviews will belong to a highly motivated group of PCPs likely more open towards innovations. We try to approach this potential bias by sampling PCPs from various age groups, gender, urban and rural regions and both having and not having been exposed to POC-PCT in the past. Also, we offer onsite and virtual interviews to not only include technologically inclined PCPs. In the workshops, there is a risk that the ideas of more experienced, more extroverted and more technology-literate participants are over-represented. We try to mitigate this by facilitating workshops by experienced members of the implementation science team and by guiding the participants. Second, there is the risk that the selected implementation strategies do not address the needs of every individual stakeholder in the complex system. By including a broad range of purposefully selected stakeholders in most of the study phases, we still believe that the needs of the majority will be addressed. Last, for the ImpPro study, we will define the final implementation strategies before the study begins and will not have the opportunity to pilot test them extensively. Likely, the selected implementation strategies will still benefit from further refinement—in agreement with the cyclical process of the double diamond design process. We thus plan to evaluate not only the clinical effectiveness of the POC-PCT delivered via the selected strategies but also to investigate implementation outcomes such as feasibility and penetration during the ImpPro trial. This interpretive formative evaluation allows further refinement of the implementation strategies for potential implementation of POC-PCT outside of the ImpPro study.^32^

In conclusion, we present the methods of a novel, rigorous four-phase process for developing a tailored, multicomponent implementation strategy to enhance the adoption, implementation and sustained utilisation of POC-PCT in the Swiss primary care setting. POC-PCT holds promise for reducing inappropriate antibiotic prescriptions in patients with lower respiratory tract infections. The planned publications of the results of our context analysis, the strategy development, as well as the hybrid effectiveness and implementation trial will inform about the success (or non-success) of our research. This is important, as—although our methods are based on well-established theories and frameworks and follow the sequence of identifying determinants and linking strategies to them—the detailed processes are novel and thus not proven to be successful. If successful, we believe that the methods we have described in detail here, or variations thereof, could be replicated to facilitate projects facing similar challenges.

## Ethics and dissemination

Based on the Swiss law for research on humans, the study was waived from the necessity for formal evaluation from the Cantonal Ethics Committee (Req-2023–00392). All interview and focus group participants in this research will be provided with an information sheet about the study, the activities they are asked to be involved in, the data handling procedure and an assurance that participation is voluntary. For interviews including audio/video recording, written informed consent from all interview partners will be obtained. The results of our study will be disseminated within the ImpPro study team and associated groups, for example, the ASAP (AMS in ambulatory care platform) group, published in peer-reviewed scientific journals and presented at national and international congresses. Data will be presented in a way that interview participants cannot be identified.

## supplementary material

10.1136/bmjopen-2024-091285 online supplemental file 1
