# Supplementary material for: Systematic method for developing tailored strategies for implementing point-of-care procalcitonin testing to guide antibiotic prescribing in Swiss primary care: a protocol for a mixed-methods participatory approach
Source: BMJ Open. 2025 Mar 5;15(3):e091285. doi: 10.1136/bmjopen-2024-091285 (PMC11883602; doi:10.1136/bmjopen-2024-091285)
Supplement: online supplemental file 1 [file bmjopen-15-3-s001.docx]

# Interview Questions for PCPs

Opening question: **Could you briefly introduce yourself and the practice you are working in?**

Practice workflow: **Imagine you're seeing a patient with an acute respiratory tract infection. Could you explain to us how the consultation with this patient proceeds, starting with the initial request for an appointment?**

| **CFIR Domain/Construct** |  |
| --- | --- |
| Individual (characteristics) & Inner Setting (tension for change) | **Do you perceive a need for POC-PCT testing?** |
| Inner setting (compatibility) | **If you think about the POC-PCT, how do you think it would fit into your practice workflow? And the patient population you serve?** |
| Innovation (evidence strength and quality) | **What type of evidence is needed to support the use of POC-PCT to guide antibiotic prescriptions?** |
| Innovation (relative advantage) | **How would you compare CRP and PCT testing with each other?**   - What are advantages or disadvantages for either of them? |
| Outer setting (Policies and laws) & Innovation (source) | **Where would you expect to hear about the recommendation to use POC-PCT?** |
| Outer setting (Policies and laws) & Innovation (source) | **Which organization or group has the most influence on you when it comes to adopting new things (innovations)?** |
| Inner setting (Access to knowledge and information) & outer setting (partnership and connections, policies and laws) | **Where / from whom would you (expect to) get information about how and when to use POC-PCT from?** |
| Outer setting (external pressure) | **How would your colleagues' choice influence your decision to implement POC-PCT?** |
| Innovation (relative advantage) | Please see this list of antibiotic stewardship interventions, POC-PCT testing is one of them.  **What are the benefits and disadvantages of POC-PCT in comparison to the other interventions?** |
| Inner setting (culture) | **Generally, to what extent are new ideas adopted and taken up in your practice?** |
| Implementation Process | **How would the implementation of POC-PCT best happen in the French Speaking part of Switzerland? What steps should be taken and by whom?** |
| Wrap up | **Is there anything you think we should know but haven't asked?** |
