# Supplementary material for: Systematic method for developing tailored strategies for implementing point-of-care procalcitonin testing to guide antibiotic prescribing in Swiss primary care: a protocol for a mixed-methods participatory approach
Source: BMJ Open. 2025 Mar 5;15(3):e091285. doi: 10.1136/bmjopen-2024-091285 (PMC11883602; doi:10.1136/bmjopen-2024-091285)
Supplement: Supplementary file 2 [file bmjopen-2024-091285.reviewer_comments.pdf]

PEER REVIEW HISTORY

BMJ Open publishes all reviews undertaken for accepted manuscripts. Reviewers are asked to complete a checklist review form and are provided with free text boxes to elaborate on their assessment. These free text comments are reproduced below.

ARTICLE DETAILS

Title (Provisional)

A Systematic Method for Developing Tailored Strategies for Implementing Point-of-Care Procalcitonin Testing to Guide Antibiotic Prescribing in Swiss Primary Care: A Protocol for a Mixed Methods, Participatory Approach

Authors

Wolfensberger, Aline; Gendolla, Sophie; Dunaiceva, Jelena; Plüss-Suard, Catherine; Niquille, Anne; Nicolet, Anna; Marti , Joachim; Powell, Byron J; Naef, Rahel; Boillat-Blanco, Noémie; Mueller, Yolanda; Clack, Lauren

---

VERSION 1 - REVIEW

**Reviewer**	**1**
**Name**	**Avent, Minyon**
**Affiliation**	**University of Queensland, UQCCR**
**Date**	**09-Oct-2024**
**COI**	**None**

Thank you for asking me to review the protocol 'Developing tailored strategies to implement point-of-care procalcitonin-testing for guiding antibiotic prescription in Swiss primary care – a mixed-methods participatory implementation research protocol'.

This protocol has been well written and includes the appropriate study design. I have no additional comments to add.

**Reviewer**	**2**
**Name**	**Huang, Eileen**
**Affiliation**	**Vanderbilt University Medical Center**
**Date**	**06-Nov-2024**
**COI**	**None**

In this paper, the authors aim to develop a protocol for implementing POC-PCT in French-speaking primary care settings in Switzerland using the CFIR framework. The paper describes

the method for conducting a formative evaluation to understand the barriers and facilitators of the implementation and to identify potential implementation strategies.

Suggestions:

1. For the title, consider replacing “implementation protocol” with “formative evaluation.” This is not an implementation protocol since several key elements of a typical implementation protocol are missing. For instance, the authors did not include specific implementation strategies, as those still need to be identified. Additionally, the logic model has yet to be developed, and metrics used to monitor the progress of implementation are not defined.

2. The evidence for using POC-PCT to reduce antibiotic prescriptions in patients with clinical pneumonia is weak, especially in French-speaking primary care settings in Switzerland. The key assumption made in the paper is that POC-PCT is effective at reducing antibiotic prescriptions in patients. However, the one reference (Ref. 3) shows that the effect is not significant, with a 26% reduction and a 95% CI that includes 0% (41% to -10%). It is unclear if POC-PCT is significantly effective at reducing antibiotic prescriptions in French-speaking primary care settings in Switzerland. Also, Reference 3 is only the protocol; it does not include the results. Please use the correct reference that includes the study results. Also, consider adding robust evidence/references to support that POC-PCT is effective and that implementing POC-PCT is crucial to reducing antibiotic prescriptions in patients with clinical pneumonia in French-speaking primary care settings in Switzerland.

VERSION 1 - AUTHOR RESPONSE

Reviewer: 1

Thank you for asking me to review the protocol 'Developing tailored strategies to implement point-of-care procalcitonin-testing for guiding antibiotic prescription in Swiss primary care – a mixed-methods participatory implementation research protocol'.

1. This protocol has been well written and includes the appropriate study design. I have no additional comments to add.

    - *We thank reviewer 1 for the positive feedback. We appreciate the acknowledgment of the study design and the protocol's quality.*

Reviewer: 2

Comments to the Author:

In this paper, the authors aim to develop a protocol for implementing POC-PCT in French-speaking primary care settings in Switzerland using the CFIR framework. The paper describes the method for conducting a formative evaluation to understand the barriers and facilitators of the implementation and to identify potential implementation strategies.

2. For the title, consider replacing "implementation protocol" with "formative evaluation." This is not an implementation protocol since several key elements of a typical implementation protocol are missing. For instance, the authors did not include specific implementation strategies, as those still need to be identified. Additionally, the logic model has yet to be developed, and metrics used to monitor the progress of implementation are not defined.

   - *We thank Reviewer 2 for this comment. As correctly noted, this protocol focuses on the methods used to tailor the implementation strategy and does not yet detail the implementation strategies, logic models, or metrics for evaluation. To clarify this for the reader, we have revised the title as follows:*

   - <u>A Systematic Method for Developing Tailored Strategies for Implementing Point-of-Care Procalcitonin Testing to Guide Antibiotic Prescribing in Swiss Primary Care: A Protocol for a Mixed Methods, Participatory Approach</u>

     (Old Title: Developing tailored strategies to implement point-of-care procalcitonin-testing for guiding antibiotic prescription in Swiss primary care – a mixed-methods participatory implementation research protocol)

3. The evidence for using POC-PCT to reduce antibiotic prescriptions in patients with clinical pneumonia is weak, especially in French-speaking primary care settings in Switzerland. The key assumption made in the paper is that POC-PCT is effective at reducing antibiotic prescriptions in patients. However, the one reference (Ref. 3) shows that the effect is not significant, with a 26% reduction and a 95% CI that includes 0% (41% to -10%). It is unclear if POC-PCT is significantly effective at reducing antibiotic prescriptions in French-speaking primary care settings in Switzerland. Also, Reference 3 is only the protocol; it does not include the results. Please use the correct reference that includes the study results. Also, consider adding robust evidence/references to support that POC-PCT is effective and that implementing POC-PCT is crucial to reducing antibiotic prescriptions in patients with clinical pneumonia in French-speaking primary care settings in Switzerland.

- *We thank the reviewer for pointing out the reference to the study protocol rather than the study results. We have made the necessary correction. The correct reference in the reference section is (not in track change due to technical reasons):*
  <u>Lhopitallier, L., et al., Procalcitonin and lung ultrasonography point-of-care testing to determine antibiotic prescription in patients with lower respiratory tract infection in primary care: pragmatic cluster randomised trial. BMJ, 2021. 374: p. n2132.</u>

- *Now reference 3 correctly refers to the results of the Ultra-Pro trial, the predecessor trial for the ImpPro study this protocol describes the methods to come up for the implementation strategies for.*
- *The Ultra-Pro trial demonstrated a statistically significant 26% reduction in antibiotic prescriptions (cluster-corrected difference −0.26 (95% CI −0.41 to −0.10)). This confidence interval does not contain 0. In addition, we consider this reduction clinically relevant, particularly in a low-prescription country like Switzerland.*
- *As the focus of this protocol is the development of the implementation strategy for the ImpPro trial (a hybrid effectiveness-implementation trial), we prefer not to include additional references supporting the clinical effectiveness of POC-PCT.*

VERSION 2 - REVIEW

**Reviewer**	**1**
**Name**	**Avent, Minyon**
**Affiliation**	**University of Queensland, UQCCR**
**Date**	**17-Dec-2024**
**COI**	

Thank you for asking me to review the protocol 'Developing tailored strategies to implement point-of-care procalcitonin-testing for guiding antibiotic prescription in Swiss primary care – a mixed-methods participatory implementation research protocol'.

The authors have addressed all the comments of the reviewers.

**Reviewer**	**2**
**Name**	**Huang, Eileen**
**Affiliation**	**Vanderbilt University Medical Center**
**Date**	**23-Jan-2025**
**COI**	

Thanks for updating your manuscript based on the feedback.
